# A profile of teen smokers who volunteered to participate in school-based smoking intervention

**DOI:** 10.1186/1617-9625-4-6

**Published:** 2008-08-05

**Authors:** Kimberly Horn, Geri Dino, Steven A Branstetter, Jianjun Zhang, George Kelley, N Noerachmanto, Cindy Tworek

**Affiliations:** 1 Translational Tobacco Reduction Research Program, Mary Babb Randolph Cancer Center and Prevention Research Center, West Virginia University, PO Box 8110, Morgantown, WV 26506-8110, USA; 2 Department of Community Medicine, West Virginia University, PO Box 9190, Morgantown, V 26506-9190, USA; 3 Department of Psychology, West Virginia University, PO Box 6040, Morgantown, WV 26506-6040, USA; 4 Department of Pharmaceutical Systems and Policy, West Virginia University, PO Box 9510, Morgantown, WV 26506-9510, USA

## Abstract

**Objectives:**

Although a number of population-based studies have examined the characteristics of teens who attempt to quit smoking, few have identified the characteristics of youth who participate in structured cessation interventions, particularly those with demonstrated effectiveness. The purpose of the present study is to describe the sociodemographic and smoking-related characteristics of teen smokers who participated in the American Lung Association's Not On Tobacco (N-O-T) program, spanning eight years. N-O-T is the most widely used teen smoking cessation program in the nation.

**Methods:**

Drawn from multiple statewide N-O-T studies, this investigation examined data from 5,892 teen smokers ages 14–19 who enrolled in N-O-T between 1998–2006. We demonstrate similarities and differences between N-O-T findings and existing data from representative samples of US teen smokers where available and relevant.

**Results:**

N-O-T teens started smoking earlier, were more likely to be poly-tobacco users, were more dependent on nicotine, had made more previous attempts to quit, and were more deeply embedded in smoking contexts than comparative samples of teen smokers. Additionally, N-O-T teens were moderately ready to quit smoking, believed important people in their lives would support their quit efforts, yet had deficits in their confidence with quitting.

**Conclusion:**

This profile of N-O-T teens can guide efforts for targeted recruitment strategies to enhance intervention reach for teen smoking cessation. Findings provide guidance for marketing and recruitment efforts of intensive, school-based cessation interventions among established teen smokers, particularly those who want to quit. Study results may shed light upon who is and is not enrolling in N-O-T.

## Background

Nearly one quarter of US high school teens currently smoke[1] and most will become adult smokers. The health and economic burden of smoking is staggering. Over 6.4 million of today's current teen smokers will eventually die of smoking-related diseases. Between 1995 and 1999, smoking was responsible for over 440,000 US deaths per year and cost the nation over $150 billion in annual health-related economic losses. Of these economic losses, $81.9 billion were related to mortality-related productivity[2]. With almost 4,000 new youth initiating smoking each day, there is an unequivocal need for medical and public health communities to identify and disseminate evidence-based interventions targeted to teens who smoke and who are motivated to quit. Research has shown that the majority of adolescent smokers want to quit,[1,3] but most spontaneous attempts end in failure[3]. Some teen smokers have both access to, and the willingness to join, a teen cessation program. However, many teens who may benefit from these programs are unaware that the programs exist. This begs the question: How can we increase teens' utilization of effective smoking cessation programs?

Although some research studies relate indirectly to this question, many critical factors remain unaddressed. For instance, numerous epidemiological studies have delineated the psychosocial predictors of smoking cessation among youth and young adults [4-6]. To date, these types of population-based studies have not examined the characteristics of teens who specifically attempt quitting through structured cessation interventions. In addition, teen smoking cessation investigations have focused primarily on efficacy[5,6], leaving unanswered questions regarding intervention reach, access, and utilization.

An initial step to increasing utilization of effective interventions is to determine the profile of teen consumers of those interventions. Clearly, numerous factors could comprise a composite profile of teen smokers. However, it is important that descriptive studies examine factors demonstrated as related to teen smoking behavior. As supported in the literature, some of these factors include (1) demographic factors such as age, race, gender, and socioeconomic status [7-9]; (2) smoking history and current smoking patterns [9-11], such as weekday and weekend cigarette consumption [11-15]; and (3) readiness to quit smoking[5,9,16-18]. A profile of current teen smokers willing to join accessible and proven cessation interventions may provide crucial information for the development and evaluation of recruitment strategies. Effective recruitment will increase intervention reach.

An extensive literature review revealed only two studies that specifically explored the characteristics of teens who participate in tobacco cessation interventions. One intervention study was part of the two-phased Helping Young Smokers Quit (HYSQ) initiative[18]. Phase II of this initiative , includes a longitudinal evaluation of 42 promising programs and the youth who participate in them. The analytic goal of the HYSQ multilevel investigation is to uncover relationships between "aggregated program attributes and youth participant outcomes." The HYSQ study findings may provide information on programs with varying degrees of effectiveness and dissemination potential. In fact, initial results indicated that the majority of participants did not change their smoking patterns, suggesting limited intervention efficacy when participant data across all cessation programs were collapsed. Unfortunately, the heterogeneity of programs included in that study makes it highly unlikely that any definitive conclusions can be made regarding the users of particular types of programs or interventions, most notably, those with previously established effectiveness.

The other study, reported by Barker and colleagues,[19] presents key findings from the National Youth Smoking Cessation Survey (NYSCS), which is a two-year longitudinal epidemiologic study of lifetime use of smoking cessation methods among teen and young adult smokers 16 to 24 years. This study included a random sample of 2,582 youth and young adult smokers who had tried to quit. Respondents answered questions about cessation methods, including knowledge, perceived availability, and use of assisted and unassisted quitting methods. The authors reported that adolescent and young adult smokers who tried quitting were more likely to use unassisted quitting methods than assisted quitting methods. Differences in utilization of cessation methods by gender were also noted where females were more likely to seek help from health professionals, quit with a friend, and use self-help cessation materials, but were less likely to use nicotine gum. Males were more likely to exercise or switch to smokeless tobacco or other tobacco products as cessation strategies. While this study provides descriptive findings related to utilization of cessation methods among a nationally representative random sample of youth and young adult smokers, it does not provide detailed information with respect to a single smoking cessation program targeted to reach established but motivated adolescent smokers.

The present investigation provides a focused exploration of years of data from a single, evidence-based teen smoking cessation program delivered to diverse youth in a variety of settings. The findings of this study compliment both the HYSQ and NYSCS studies and enhance the knowledge about how to increase consumer (i.e., teen) demand for evidence-based teen cessation services. To address this current research gap in teen smoking cessation, the present study generated a profile of baseline characteristics from seven years of research on the American Lung Association's Not-On-Tobacco (N-O-T) program. N-O-T is a 10-session theory-based program delivered by trained facilitators[4,20,21]. N-O-T was developed specifically for adolescents, with program content and methods geared toward relevant developmental issues (e.g., coping skills, decision making, autonomy, peer pressure, and so on). N-O-T sessions cover topics such as the following: myths and realities about smoking; understanding addiction; health and social impacts of smoking on mind and body; sharing the quitting experience; avoiding risky situations and relapse; stress management; handling pressure from friends and family; understanding tobacco advertising; and staying committed to quitting. Effectiveness studies on the N-O-T program reveal end-of-program intent-to-treat quit rates between 15% and 19% for 1998–2003 [20]. These rates are among the highest quit rates reported in the literature. The state-of-the-art in teen smoking cessation shows that a handful of programs or strategies demonstrate meaningful results [14,18,22]. A systematic review of two and a half decades of youth tobacco-use cessation found an average quit rate of 14%[14]. Only two programs receive praise as evidence-based programs: N-O-T and Project EX [14,18,22].

N-O-T's success has resulted in federal recognition as a Substance Abuse and Mental Health Administration (SAMHSA) evidence-based Model Program, a National Cancer Institute (NCI) Research Tested Intervention Program, and an Office of Juvenile Justice and Delinquency Prevention (OJJDP) Model Program. Moreover, a cost-effectiveness analysis comparing N-O-T to a minimal-contact brief intervention indicated that N-O-T is a highly cost effective option for school-based tobacco intervention[21]. Importantly, N-O-T has been disseminated in 48 states, is currently the most widely used smoking cessation program in the nation, and has been rigorously assessed for adoptability. In fact, a recent survey of youth smoking cessation programs found N-O-T to be the most widely used intervention in the nation[18]. The study purpose is to identify the sociodemographic and smoking-related characteristics of teen smokers who participated in the N-O-T program. Findings will help elucidate the profile of young smokers that have participated in N-O-T and inform the literature concerning characteristics related to young smokers' utilization of school-based smoking cessation programs

## Methods

### Participants

Data for the present analysis were taken from a five-state sample of teen smokers (n = 6,635) who voluntarily participated in either matched-design or field studies of the N-O-T program between 1998 and 2006 [20]. As a part of larger statewide N-O-T evaluation studies, participants represented youth from FL, NC, NJ, WI, and WV. Researchers collected participant data prior to the onset of intervention, across all evaluations. Because N-O-T is designed for regular smokers, likely to be addicted, eligible participants had to smoke ≥ 5 cigarettes per day. They also had to be enrolled at the school sites. The N-O-T program is designed for 14- to 19-year-old regular smokers; therefore, analyses included only participants within that age range. Given these criteria, the final overall sample included 5,892 teens. Importantly, not all study instruments were administered in all studies; therefore, the sample size analyzed in the present study varied depending on the questionnaire(s) or variables being examined. All study procedures received Institutional Review Board approval.

### Data sources

We identified 24 baseline variables consistently demonstrated in the literature as significantly related to teen smoking to assure that relevant characteristics were assessed (refer to Table 1). Specifically, we assessed variables related to basic demographics,[5,11,13,23] smoking history,[3,5,13,14] and intervention readiness[15,23-25]. Table 1 indicates the data sources and response options used for the selected variables. Two study variables warrant additional details beyond what is provided in Table 1 – "nicotine dependence" and "stages of change." The study assessed *nicotine dependence *using the widely accepted Fagerstrom Tolerance Questionnaire (FTQ)[26]. Throughout the years, FTQ developers made various modifications to adapt its use for teens, validating the FTQ and utilizing it to measure nicotine dependence among adolescent smokers. Thus, over time, our N-O-T studies used two different versions of the FTQ instrument. The first version was the original eight-item questionnaire developed by Fagerstrom, with a score range of 0–11[24,26]. The second version was a seven-item revision of the original FTQ[24,25]specifically developed for teens; with a score range of 0–9. The modified FTQ eliminated one item related to cigarette brand. Both instruments rendered a sum score of nicotine dependence ranging from no dependence to high dependence. To assure that our data were uniform for analyses, we collapsed data from both versions of the instrument into three levels of nicotine dependence (low, moderate, and high). We then merged all data into these appropriate standardized subgroups. Consistent with FTQ scoring recommendations, scores were 0–2 (low dependence or no dependence), 3–5 (moderate dependence), and 6 or higher (high dependence).

**Table 1:** Critical Study Variables

**Variables**	**Response Options**
***Basic demographics:***	
Age	Ages 14–19
Gender	Male or Female
Grade	Grades 7–12
Living arrangements	1) Both parents, 2) father only, 3) mother only, 4) grandparent, 5) father and step mother, 6) mother and step father, 7) other
Race	1) White, 2) African American, 3) American Indian,4) Asian American, 5) Hispanic, 6) Native Hawaiian, 7) other/multi racial
	
***Smoking history:***	
Age of first try	1) <= 9 years; 2) 10 – 14 years; 3) > 14 years
Other tobacco use: smokeless	1= No; 0 = Yes (past 30 days)
Other tobacco use: cigars	1 = No; 0 = Yes (past 30 days)
Other tobacco use: nicotine replacement	1 = No; 0 = Yes (past 30 days)
Previous quit attempts	1 = No; 0 = Yes (ever attempted)
Smoking status of parents	1 = No; 0 = Yes (current use)
Smoking status of siblings	1 = No; 0 = Yes (current use)
Smoking status of friends	1 = No; 0 = Yes (current use)
Smoking status of boy/girlfriend	1 = No; 0 = Yes (current use)
Number of Cigarettes smoked per weekday	Cigarettes per day (min. 1 – max. 90)
Number of Cigarettes smoked per weekend day	Cigarettes per day (min.1 – max. 90)
Level of nicotine dependence	1) Low; 2) Moderate; 3) High
	
***Intervention Readiness:***	
Motivation to quit	1) None; 2) Low; 3) Medium,; 4) High; 5) Very High
Confidence to quit	1) None; 2) Low; 3) Medium,; 4) High; 5) Very High
Stages of change	1) Do not plan to quit in next 6 months; 2) plan to quit in next 6 months; 3) plan to quit in next 30 days; 4) made a serious quit attempt in past 6 months; 5)quit less than 6 months ago
Perceived parents support to quit or reduce	1 = No; 0 = Yes
Perceived siblings support to quit or reduce	1 = No; 0 = Yes
Perceived friends support to quit or reduce	1 = No; 0 = Yes
Perceived boy/girlfriends support to quit or reduce	1 = No; 0 = Yes

As supported by several classic references in the literature,[7,11,24,27-29] our "intervention readiness" construct broadly reflected psychosocial factors that may influence a teen's readiness to quit smoking. These factors included motivation to quit, confidence to quit, and stages of change (refer to Table 1). It is essential to explore readiness because smoking cessation interventions are less likely to achieve success with smokers who are not ready to quit[28]. We used *stages of change *[25] as part of the readiness construct rather than a stand alone measure of readiness, as is often used. As previously noted, research shows that many variables influence readiness. Readiness is not a linear process and may involve fluid movement in and out of certain stages at various points in time[25]. Our construct reflected participants' state of readiness at baseline, a fixed point in time. Our stages of change variable included response options suggested by Prochaska and colleagues in the transtheoretical model[25]. The five stages of change were precontemplation, contemplation, preparation, action, and maintenance (scaled from 1–5, respectively). The study defined *precontemplation *as "Do not plan to quit in next 6 months"; *contemplation *as "plan to quit in next 6 months"; *preparation *as "plan to quit in next 30 days"; *action *as "made a serious quit attempt in past 6 months"; and *maintenance *as "quit less than 6 months ago." The Transtheoretical Model posits that successful behavior change requires moving through these five stages.

### Analyses

As shown in Table 1, we grouped the 24 baseline variables into three different categories: ***basic demographics***, which included age, gender, grade, and living arrangements; ***smoking history***, including age of trying first cigarette, other tobacco use, number of cigarettes smoked per day on weekdays and weekends, nicotine dependence level, previous quit attempts, and smoking status of parents and friends; and ***intervention readiness***, which included smoking-related psychosocial variables such as motivation and confidence to quit, and stages of change. The variable values are shown in Table 1. We conducted frequency analyses for all variables, and mean values were calculated, as appropriate. Because some research shows that the stages of change construct alone does not capture the full state of readiness,[29-32] for some analyses, we collapsed our readiness factors to form an aggregate readiness to quit smoking score. Using the values shown in Table 1, the overall readiness score ranged between 3 and 15. In addition to basic descriptive statistics, the present study utilized Pearson's product moment and point-biserial correlations and multiple linear regression with backward elimination. Given the multiple comparisons and analyses made in the present study, we controlled for Type I error using a method called the False Discovery Rate (FDR). In contrast to methods that are designed to control for any false positive (e.g., Bonferroni methods), the FDR method controls for the proportion of false positive that could be expected given the total number of tests. The FDR method has the advantage of increased power, efficiency, and less risk of Type II errors than Bonferroni procedure. FDR values can be interpreted as the confidence in that prediction; for example, a FDR value of .20 would suggest that no more of 20% of the findings are erroneous. Whereas no standard has been set for a cut-off for "acceptable" FDR levels, we have used a conservative level of .05. For ease of presentation, all results presented as significant at a level of *p *< .05 were found to have FDR values < .05. Cases where *p *values of < .05 had FDR values *greater *than .05 are specifically noted.

## Results

### Basic demographics

Among all participants, there were more females than males; the mean age was 16 (SD = 1.15), and grades 9–12 were almost equally represented. Almost half (43.90%) of the teens lived with both biological parents. A majority of teens were white (74.75%). Refer to Table 2 for additional descriptive data.

**Table 2:** Summary of Critical Characteristics – Basic Demographics

**Items**	**N**	**%**
**Gender**		
Female	3284	55.76
Male	2606	44.24
Total	5890	100.00
**Age (mean ± SD)**	**16.05 ± 1.15**	
14.00	514	8.72
15.00	1479	25.10
16.00	1787	30.33
17.00	1482	25.15
18.00	568	9.64
19.00	62	1.05
Total	5892	100.00
**Grade**		
7	14	0.24
8	72	1.24
9	1482	25.46
10	1653	28.40
11	1540	26.46
12	1059	18.20
Total	5820	100.00
**Living with**		
Father and Mother	827	43.90
Father only	95	5.04
Mother only	355	18.84
Grandparent	71	3.77
Father and Stepmother	88	4.67
Mother and Stepfather	337	17.89
Other	1111	5.89
Total	1884	100.00
**Race**		
White	4234	74.75
African American	214	3.78
American Indian	79	1.39
Asian American	104	1.84
Hispanic	711	12.55
Native Hawaiian	19	0.34
Other	303	5.35
Total	5664	100.00

Note: SD = standard deviation

### Smoking history

Most (74.27%) participants started smoking cigarettes between the ages of 10 and 14 (Mean = 12.14, SD = 2.32). In the past 30 days (current use), only 12.65% had also used smokeless tobacco, but 44.75% had smoked cigars. Very few teens (5.26%) had ever used any type of nicotine replacement. Among all youth, most (78.18%) previously made at least one attempt to quit smoking cigarettes, despite unfavorable smoking environments where 65.27% of parents, 55.19% of siblings, and 93.46% of close friends were smokers. Our study showed that youth smoked more than half a pack of cigarettes (Mean = 12.03, SD = 10.45) per day on weekdays and almost one pack (Mean = 18.29, SD = 12.68) per day on weekends, upon program enrollment. Participants had moderate to high FTQ nicotine dependence scores. Specifically, scores were 0–2 (low dependence or no dependence, 10.98%), 3–5 (moderate dependence, 30.03%), and 6 or higher (high dependence, 58.99%). See Table 3.

**Table 3:** Summary of Critical Characteristics – Smoking Status and History

**Items**	**N**	**%**
**Age of first try (mean ± SD)**	**12.14 ± 2.32**	
<= 9 years	764	12.97
10 – 14 years	4376	74.27
> 14 years	752	12.76
Total	5892	100.00
**Other tobacco use:**		
Smokeless – Yes	75	12.65
Cigars – Yes	264	44.75
Nicotine replacement – Yes	22	5.26
Level of nicotine dependence*		
Low	72	10.98
Moderate	197	30.03
High	387	58.99
Total	613	100.00
**Previous quit attempts **– Yes	4537	78.18
**Smoking status**		
Parents smoking – Yes	1492	65.27
Sibling smoking – Yes	1068	55.19
Friends smoking – Yes	2143	93.46
Boy/Girlfriend smoking – Yes	149	59.84
**Number smoked per weekday (mean ± SD)**	**12.03 ± 10.45**	
**Number smoked per weekend day (mean ± SD)**	**18.29 ± 12.68**	

Note: SD = Standard Deviation; *FTQ standard scoring used.

### Intervention readiness

A majority of the participants had moderate to very high levels of motivation to quit smoking (Mean = 3.19, SD = 0.89); confidence levels were slightly lower (Mean = 3.00, SD = 0.89). Analyses of the stage of change variables (Mean = 2.43, SD = 0.95) showed that 51.28% of youth planned to quit smoking in the next 6 months. Teen scores on overall readiness were 8.42 (SD = 2.02). Refer to Table 4.

**Table 4:** Summary of Critical Characteristics – Intervention Readiness

**Items**	**N**	**%**
**Motivation to quit (mean ± SD)**	**3.19 ± 0.89**
None	26	2.45
Low	178	16.78
Moderate	503	47.41
High	276	26.01
Very high	78	7.35
Total	1061	100.00
**Confidence to quit (mean ± SD)**	**3.00 ± 0.89**
None	37	3.87
Low	214	22.38
Moderate	474	49.58
High	172	17.99
Very high	59	6.17
Total	956	100.00
**Stages of change (mean ± SD)**	**2.43 ± 0.95**
Do not plan to quit smoking in the next 6 months	168	11.91
Plan to quit smoking in the next 6 months	723	51.28
Plan to quit within the next 30 days	301	21.35
Made serious quit attempts in the past 6 months	179	12.70
Quit less than 6 months ago	39	2.77
Total	1410	100.00
***Intervention readiness total score *(mean ± SD)**	**8.42 ± 2.02**

Note: SD = Standard Deviation.

### Correlations among basic demographics, smoking history, and intervention readiness

We examined relationships across variables, with the assumption that certain demographic and smoking history variables would be related to intervention readiness. None of the basic demographic factors were related to intervention readiness. Among the smoking history variables, only amount of smoking was significantly related to overall readiness (i.e., the aggregate readiness score). Specifically, Pearson's correlation showed that the higher the number of cigarettes smoked per day on weekdays and weekend days, the lower the readiness scores (weekday: *r (266) *= -0.19, *p *= 0.01; weekend: *r (267) *= -0.20, *p *< 0.001).

Although not related to readiness, there were other Pearson correlations worthy of discussion. Analysis showed positive associations between number of cigarettes smoked per weekday, weekend day and level of nicotine dependence (weekdays: *r (669) *= 0.47, p < 0.001; weekends: *r (669) *= 0.47, p < 0.001) and a negative correlation with age of first try (weekdays: *r (5750) *= -0.15, p < 0.001; weekends: *r (5726) *= -0.17, p < 0.001). Age of first try was negatively correlated with both nicotine dependence (*r (654) *= -0.11, *p *= 0.004), and parent smoking status (*r (2251) *= -0.16, p < 0.001). Next, motivation to quit was positively correlated with confidence to quit (*r (949) *= 0.54, p < 0.001). Finally, the more support teens perceived from parents, siblings, and close friends, the higher was their motivation to quit (Parental support: *r (423) *= 0.13, *p *= 0.01; Sibling support: *r (380) *= 0.11, *p *= 0.02; Friend support: *r (425) *= 0.20, *p *< 0.001) and was their confidence to quit (Parental support: *r (423) *= 0.10, *p *= 0.039; Sibling support: *r (380) *= 0.10, *p *= 0.04; Friend support: *r (425) *= 0.13, *p *= 0.08).

### Explanation of variance in smoking variables and intervention readiness

To understand the relation between variables, we conducted multiple linear regression analyses with backward elimination. This method begins with a saturated model with all relevant predictors entered simultaneously. Next, each predictor is evaluated, one at a time, for the reduction of the overall model *R*^2 ^that would result from that variable's deletion from the model. Variables removed resulting in an insignificant reduction in *R*^2 ^are then deleted. A final model results when no further predictor variable can be deleted from the model.

For these regression analyses, we entered the demographic, smoking history, and intervention readiness variables into the model as predictors. For the purposes of the regression analyses, categorical variables (race, living arrangement) were dummy coded and each variable was entered separately. Additionally, to examine the potential influence of the state in which the participants lived, we dummy coded state (NJ, WV, NC, WI, FL) and included the resulting variables in the analysis. This resulted in a total of 32 predictor variables. A power analysis demonstrated that the current sample size (N = 5,892) was sufficient to detect a very small effect size (*f*^2 ^= .01) with power of .99, α = .01 with 32 predictors in the model.

### Weekday smoking frequency

For the initial model, weekday smoking was removed from the list of predictor variables and added as the dependent variable. The overall initial model was significant, *F (28, 144) = 7.57, p *< .001, and accounted for over half the variance in weekday smoking, adjusted *R*^2 ^= .52. Likewise, the final model was significant, *F (6, 166) = 36.66, p *< .001 and explained over half the variance in weekday smoking, adjusted *R*^2 ^= .55. The final model eliminated 25 of the initial predictor variables, with only the race ("other race" dummy-coded variable; *p *= .008), gender (*p *= .001), use of cigars (*p *= .02), previous quit attempts (*p *= .03), number of cigarettes smoked on weekends (*p *< .001) and nicotine dependence (*p *= .001) remaining as significantly associated with weekday smoking. In the final model, "other" race, male gender, nicotine dependence level, having had a previous quit attempt, use of cigars and number of cigarettes smoked on weekends were all associated with an increase in weekday smoking.

### Weekend smoking frequency

Similar to the previous model, the initial model examining weekend smoking as the dependent variable was significant, *F (28, 144) = 7.11, p *< .001, and accounted for half the variance in weekend smoking, adjusted *R*^2 ^= .50. The final model was also significant, F *(7, 165) = 28.45, p *< .001, and accounted for just over half the variance in weekend smoking, adjusted *R*^2 ^= .53. The final model eliminated 24 of the initial predictor variables leaving race ("other race" dummy-coded variable; *p *= .45), living with mother and step father (*p *= .05), gender (*p *= .59), age of first tobacco use (*p *= .014), use of cigars (*p *= .005), weekday cigarette use (*p *< .001), and level of nicotine dependence (*p *= .016). In the final model, younger age of first tobacco use, cigar use, number of cigarettes smoked on weekdays and nicotine dependence were all associated with an increase in weekend cigarette smoking. In contrast to weekday smoking, the "other" race category was associated with less weekend smoking.

### Level of Nicotine Dependence

Again, the initial model examining nicotine dependence as the dependent variable was significant, *F (28, 144) = 3.48, p *< .001, and accounted for just over one-quarter of the variance in nicotine dependence smoking, adjusted *R*^2 ^= .28. The final model was also significant, *F (5, 167) = 16.10, p *< .001, and accounted for nearly one-third of the variance in nicotine dependence smoking, adjusted *R*^2 ^= .31. A total of 26 of the initial predictor variables were eliminated in the final model leaving state (Florida; *p *= .001), race ("Caucasian" dummy coded variable; *p = .094*), living with father alone *(p = .01)*, weekday smoking (*p < .001*), and weekend smoking (*p *= .032). The final model demonstrated that living with a father alone, and both weekday and weekend smoking frequency were related to nicotine dependence. In addition, being from the state of Florida was associated with lower levels of nicotine dependence.

### Motivation to quit

Both the initial model, *F (28, 144) = 4.06, p *< .001, and the final model, *F (4, 168) = 26.05, p *< .001, with motivation to quit as the dependent variable were significant. Additionally, both the initial model, adjusted *R*^2 ^= .34, and the final model, adjusted *R*^2 ^= .38, explained over a third of the variance in motivation to quit. The final model eliminated 27 of the initial predictor variables, leaving in the final model age (*p *= .08), confidence in quitting (*p *< .001), stage of change, (*p *= .004), and status of boy/girlfriend smoking (*p *= .002). The final model demonstrated that increased age, increased confidence and higher stages of change were associated with higher levels of motivation to quit. Having a boy or girlfriend who smokes was associated with less motivation to quit.

### Confidence in quitting

The initial model with confidence in quitting as the dependant variable was significant, *F (28, 144) = 4.35, p *< .001 and accounted for over a third of the variance, adjusted *R*^2 ^= .35. Likewise, the final model was significant, *F (9, 163) = 12.61, p *< .001, and accounted for 38% of the variance in confidence in quitting, adjusted *R*^2 ^= .38. See Table Y for results. In the final model, 22 of the initial predictor variables were eliminated, leaving Caucasian race (*p *= .05), Asian race, (*p *= .08), Hispanic race (*p *= .008), living with mother and step father (*p *= .06), living with other guardians (*p = .04*), use of cigars (*p *= .07), smoking status of boy/girlfriend (*p = .002*), cigarettes smoked on weekends (*p *= .03) and motivation to quit (*p *< .001). In this model, Hispanic race, use of cigars, number of cigarettes smoked on weekdays, and having a boy or girlfriend who smokes were all associated with less confidence in quitting. Living with a guardian other than parents/grandparents and higher levels of motivation to quit were associated with increased levels of confidence.

### Stage of change

For the purposes of these analyses, stage of change was treated as a continuous variable, with higher scores indicating higher or more advanced "stages" of the change. The initial model was significant, *F (28, 144) = 1.81, p *= .013 and accounted for a quarter of the variance, adjusted *R*^2 ^= .25. The final model was also significant, *F (5, 167) = 8.28, p *< .001 and accounted for 20% of the variance, adjusted *R*^2 ^= .20. The final model eliminated 28 of the initial predictors leaving only living situation (mother and stepfather; *p *= .09), grade (*p *= .03), previous quit attempts (*p *= .001), and motivation to quit (*p *< .001). In the final model, higher grade, previous quit attempts and higher motivation to quit were associated with higher stages of change.

## Discussion

Our sample reflected a diverse group of teen smokers; approximately 75% were white, followed by 12% Hispanic. The racial and ethnic percentages in our sample were equivalent to the sample of teen smokers surveyed by the HYSQ study [18,33]. Our sample initiated regular smoking at the age of 12 years. For decades, the literature indicated an US average age of smoking onset between ages 15 and 16 [1,2]. Consistencies were found among the self reported data from teens regarding their smoking histories [34]. Our sample, similar to those found by HYSQ, reported smoking onset at least three years earlier. Given a participant mean age of 16, seemingly these young intervention seekers had smoked for four to five years before they joined a cessation program.

We also found that the earlier a teen began smoking, the greater was his/her level of nicotine dependence. As expected, frequency of weekday and weekend smoking also were highly correlated with dependence [35]. The N-O-T teens were not experimental smokers – they were moderately to highly nicotine dependent. This level of dependence contrasts with data from other findings in which nearly 80% of teen smokers were classified as having low or very low levels of nicotine dependence as measured by the revised Fagerstrom Test for Nicotine Dependence[35-37]. N-O-T is designed for regular smokers, likely to be addicted, which may account for this difference [38]. Importantly, this finding validates the enrollment of N-O-T's intended population of addicted smokers, as indicated by teens' self-selection into the program.

Additionally, approximately half of the sample consisted of poly-tobacco users at baseline; cigars were the most frequently used secondary tobacco product, followed by smokeless tobacco. Our sample of teens were about 60% more likely than other teens to use smokeless tobacco and over 200% more likely to use cigars than other national samples of teen smokers[36,37]. Because N-O-T is designed for regular cigarette smokers, our studies did not enroll youth who used *only *smokeless, cigars, or any other form of tobacco. By design, participants must smoke cigarettes to be eligible for N-O-T. Our sample of N-O-T teens smoked daily, consuming at least a pack per day on weekends and just over a half a pack a day Monday through Friday. Similar to other research, the regression analyses showed that being male with advanced smoking behaviors, including poly-tobacco use, high dependence, and previous quit attempts, were associated with the highest levels of weekday smoking [39,40]. Advanced smoking behaviors, regardless of gender, were associated with heavy weekend smoking. We acknowledge a range of daily intake as suggested by the standard deviations reported in Table 3. However, the point remains that N-O-T participants smoked daily, some more heavily than others did. Living with "father only" was also predictive of dependence levels. Perhaps this is the result of lower supervision and monitoring of adolescent behavior in father-only homes, or possible exposure to parental smoking if the father is a current or former smoker. Indeed, research has found that adolescents from father-only homes engage in more alcohol and drug behaviors, as well as engage in more delinquent behaviors than those from mother-only and intact families [41-45].

High daily smoking rates among N-O-T teens also may explain higher nicotine dependence scores than found in previous teen studies. Other N-O-T studies have shown high dependence levels among participants [4,38]. In contrast, the HYSQ study found that among all US teen smokers who enrolled in cessation programs, only about half reported smoking every day in the past 30 days[18]. Generally, this suggests that a high percentage of light or experimenting smokers participated in the HYSQ study. Comparisons reveal that upon enrollment, N-O-T teens smoke with greater intensity and frequency than the general group of intervention seekers in the HYSQ sample. These teens may be more attracted to intensive programs, such as N-O-T, because they can clearly identify themselves as regular smokers with a significant enough habit to require intensive intervention. As found in other N-O-T studies, few N-O-T teens in the current sample had ever used any type of nicotine replacement. In fact, in our previous N-O-T studies, teens expressed a strong opposition for nicotine replacement[2,38]. Only 5% of our N-O-T sample reported ever using any type of nicotine replacement. In contrast, other studies have found higher usage of nicotine replacement among teen smokers [46-48]. For example, a study of Tennessee teens found that almost 17% of daily smokers reported using nicotine replacement at least once [48]. In HYSQ, 66% of teens had used some method to help them quit smoking; over 20% of those teens reported using nicotine replacement[18]. Overall, our findings on smoking history suggest that N-O-T attracts teens who are heavy smokers, highly addicted, and who are less interested in pharmaco-intervention as an aid to smoking cessation. It likely that other programs recruit or enroll light or experimenting smokers. Interestingly, a recent paper by Backinger and colleagues [49] found that cessation program recruitment and retention were higher for youth who smoke > 6 cigarettes per day. Given that our sample had been smoking for several years, was smoking nearly a pack of cigarettes a day, and was at least moderately dependent on tobacco, it is no surprise that they were not novices at quit attempts. Prior to joining N-O-T, 78.18% of our sample had made previous quit attempts. Previous studies have found that between approximately 27% and 54% of current teen smokers have made attempts to quit smoking [48,50]. More participants reported their motivation to quit as high or very high as compared with their confidence to quit. Unsuccessful previous quit attempts may explain why teen have low confidence in their abilities to quit but remain motivated. Consistent with the voluntary nature of N-O-T, enrollees were moderately motivated and moderately confident regarding their capacity to quit smoking. Most planned to quit smoking in the next 1–6 months, the general timeframe of N-O-T participation. Being older, more confident, and in higher stages of change were associated with higher motivation to quit. Having a romantic boy/girlfriend who smokes was associated with less motivation and less confidence to quit. Other factors associated with low confidence were being Hispanic, heavy weekday smoking, and cigar use.

Similar to past research with teen smokers, the heavier smokers were more nicotine dependent [29] and less ready to quit smoking[30]. Teens in higher grades, with previous quit attempts, and higher motivation were the most likely sub-group to be in advanced stages of readiness. Heavy smokers may show lower readiness because they lack confidence in their ability to quit, suggesting that cessation programs should address confidence issues at program start up, especially among addicted, heavy smokers. Success with cessation can depend on the balance between a smoker's readiness to stop smoking and his or her level of dependence on cigarettes.

Almost all N-O-T teens had significant people in their social networks who smoke – namely, parents, siblings, friends, and boy/girlfriends. Most teens perceived that friends and family would support their efforts to quit smoking. Interestingly, our participants reported that parents were less likely to support quitting than were age-mates. Ample research has found that such environmental contexts are clearly related to ongoing smoking and failed cessation attempts [28-30]. For example, age of first try was correlated with parent smoking status, suggesting that parent smoking increases the risk of early onset smoking among offspring. A related finding showed positive relations between parent smoking and sibling smoking. Decades of research in developmental psychology and social learning show that familial influences are one of the primary predictors of youth health behavior, including smoking. Despite past research highlighting peer influences on youth smoking, recent research has focused on, and identified, strong associations among family and other environmental predictors and teen smoking [44-46]. Certainly, family influences on onset and readiness to quit smoking were evident in this study. Unfortunately, our sample appeared to be deeply immersed in a smoking culture, both at home and within their peer context; they had more parents who smoke and more friends who smoke than most teen smokers. These findings have important implications for program recruitment. N-O-T teens may represent a group of "hard core" adolescent smokers who bring significant challenges to any cessation attempt. Thus, effective recruitment may require that potential N-O-T participants have face-to-face communication with facilitators and N-O-T "graduates." This type of active recruitment may be important to convey up front how the program helps youth deal with these specific barriers to quitting, such as parents and friends who smoke, high levels of daily use and nicotine addiction, as well as low confidence in quitting.

What we learned about teens who participated in N-O-T also shed light on who is not participating, such as low dependent, low frequency smokers, and late starters. More specifically, describing typical users of school-based smoking cessation programs can also help to identify groups of teens who are not utilizing services, as represented by the small percentage of adolescent and young adult smokers using assisted quitting methods in the NYSCS [19]. Future studies should determine recruitment methods necessary to engage their participation and generate demand.

## Limitations

Caution is warranted in the application of findings nationally. Results may be most applicable to states or local areas with demographically similar populations and smoking characteristics as found in our sample. Another limitation may be in using descriptive data to generalize about N-O-T participant characteristics. This study did not determine reasons for participation. The field needs additional in-depth study to address motivation or reasons for program participation. Participation may be related to intrinsic motivation or it may also be related to the tobacco control environment at state and local levels.

## Conclusion

A primary study objective was to present a profile of youth from across the US who voluntarily participated in the N-O-T program. Generally, we found that teens who sought N-O-T intervention started smoking during their preteen years, were poly-tobacco users, were highly dependent on nicotine, had made previous attempts to quit (and were not favorable toward NRT), were daily smokers, and were deeply embedded in smoking contexts.

Interestingly, we did not identify sociodemographic factors that predicted intervention readiness as measured in our study. It is possible that other, unmeasured factors influence a teen smoker's readiness to quit using a teen smoking cessation program. It is also possible that a better measure of readiness needs to be identified. However, as discussed above, we found a few interesting results when we entered the individual variables (within the construct) into the regression model. In depth research is needed to explore this issue. Although we found a few sources of comparative data for the general US teen smoking population and from other intervention seekers, it would be useful in the future to explore a cohort sample of teen smokers comprised of both intervention seekers and nonintervention seekers. The development of effective recruitment strategies for teen smoking cessation interventions will be best informed by identifying the characteristics of both treatment- and non treatment-seeking smokers. However, the importance of the current study should not be minimized. Experts have underscored the importance of recruitment. This composite profile of teen smokers who participated in N-O-T can guide efforts for targeted recruitment strategies to enhance intervention reach for N-O-T and similar programs with demonstrated efficacy. Our findings are particularly relevant to marketing and recruitment efforts of the N-O-T program, the most widely used program in the US[18] and the most widely evaluated smoking cessation program in the world[50].

## Competing interests

The authors declare that they have no competing interests.

## Authors' contributions

KH was the PI or Co-PI on all studies providing the data for this investigation. She conceived the investigation and was the primary author on the paper, overseeing all aspects of the manuscript. GD was the PI or Co-PI on all relevant studies and contributed to the writing of the analyses and discussion. SB participated in the design of the investigation and directed the statistical analysis. JZ and GK performed the statistical analyses, and reviewed the manuscript. NN prepared the analyses, tables, and reviewed the manuscript. CT reviewed, edited, and assisted in the writing of the discussion. All authors read and approved the final manuscript.
